# Associations Among Wearable Activity Tracker Use, Exercise Motivation, and Physical Activity in a Cohort of Cancer Survivors: Secondary Data Analysis of the Health Information National Trends Survey

**DOI:** 10.2196/24828

**Published:** 2021-04-12

**Authors:** Steven De La Torre, Donna Spruijt-Metz, Albert J Farias

**Affiliations:** 1 Department of Preventive Medicine Keck School of Medicine University of Southern California Los Angeles, CA United States; 2 Dornsife Center for Economic and Social Research University of Southern California Los Angeles, CA United States; 3 Department of Psychology University of Southern California Los Angeles, CA United States; 4 Norris Comprehensive Cancer Center Keck School of Medicine University of Southern California Los Angeles, CA United States

**Keywords:** mHealth, mobile health, cancer survivors, exercise, physical activity, motivation, wearable electronic devices, fitness trackers

## Abstract

**Background:**

Cancer survivors who meet physical activity (PA) recommendations (≥150 minutes of moderate-to-vigorous physical activity [MVPA] per week) experience better health outcomes. With the growing availability of wearable activity trackers (WATs), it may be easier to track PA. However, it is unknown what motivates survivors to use these devices.

**Objective:**

The aim of this study is to investigate the associations among motivations for exercise, previous WAT use for tracking a health goal or activity, and meeting the recommended amount of PA among a cohort of cancer survivors.

**Methods:**

Data on WAT users who reported having a previous cancer diagnosis were analyzed from the National Cancer Institute’s Health Information National Trends Survey 5 Cycle 3. All survivors with complete information on demographics, exercise motivations (internal guilt, external pressure, physical appearance, and exercise enjoyment), previous WAT use (yes or no), and minutes of MVPA per week (N=608) were included. Multivariate logistic regression models were used to test these associations. A separate cluster analysis was conducted to identify the profiles of exercise motivation that were associated with reporting WAT use.

**Results:**

The mean age of the cohort was 66.9 years (SD 12.1). The majority were non-Hispanic White (473/608, 78.8%) and female (322/608, 54.9%), and skin cancer was the most commonly reported diagnosed cancer (154/608, 27.8%). Survivors who reported using WATs to track a health goal or activity were 1.6 times more likely to meet MVPA recommendations than those who did not use WATs (odds ratio [OR] 1.65, 95% CI 1.03-2.65; *P*=.04). When exercise motivations were assessed independently, survivors who reported not feeling any internal guilt as an exercise motivation were 73% less likely to report having used a WAT than those who felt any internal guilt (OR 0.27, 95% CI 0.14-0.54; *P*<.001). A total of 3 distinct motivational profiles emerged from the cluster analysis. WAT users had an increased probability of membership in profile 3, which was characterized as being strongly motivated to exercise by internal guilt, physical appearance, and exercise enjoyment (OR 4.5, 95% CI 2.1-9.7; *P*<.001).

**Conclusions:**

Among this cohort, survivors who reported using WATs to track a health goal or activity were significantly more likely to report meeting PA recommendations. Survivors who reported feeling internal guilt as an exercise motivation were significantly more likely to report using WATs to track a health goal or activity. When examining clusters of motivation, survivors who reported previous WAT use were more likely to report being motivated to exercise by a mix of intrinsic and extrinsic motivations, including internal guilt, exercise enjoyment, and physical appearance. Given the health benefits of PA for cancer survivors, technology-focused interventions that use WATs and target exercise motivation may aid in cancer survivors meeting the level of recommended PA.

## Introduction

### Background

There are more than 16.9 million cancer survivors living in the United States, and this number is expected to reach more than 22.1 million by 2030 [1]. From 1997 to 2014, obesity increased more rapidly among adult cancer survivors than in the general population [2]. Furthermore, there is a higher prevalence of obesity among cancer survivors from underrepresented populations, such as Hispanics, compared with White cancer survivors [3-8]. In addition, Hispanic breast cancer survivors tend to have lower levels of physical activity (PA) than their non-Hispanic White counterparts [9]. Obesity has several negative health consequences that affect cancer survivors. Obesity puts survivors at a greater risk for cardiovascular disease, diabetes, and cancer recurrence [10-14]. In addition, accumulation of adipose tissue can inhibit effective cancer treatment [15].

PA plays an important role in reducing obesity and increasing quality of life among breast, colorectal, prostate, and multiple site cancer survivors [16-18]. PA can help reduce morbidity and mortality and alleviate the negative side effects of chemotherapy, including fatigue, nausea, disturbed sleep, decreased activity, and impaired quality of life [19-22]. Thus, guidelines from the American Cancer Society recommend that cancer survivors engage in at least 150 minutes per week of moderate-to-vigorous physical activity (MVPA) [23]. However, only 17% to 37% of breast cancer survivors in the United States adhere to these recommendations and most tend to exercise less after treatment [24-27].

Cancer survivors have unique health-related physical and psychological challenges resulting from the acute and long-term effects of cancer, including declines in physical functioning, decreased exercise motivation, and increased levels of anxiety and fatigue [28-31]. Innovative approaches are required to address these challenges. Wearable activity trackers (WATs) are promising tools for addressing these barriers. As of 2020, approximately 1 in 5 US adults (21%) say they regularly wear a smart watch or wearable fitness tracker [32]. WATs that monitor PA act as a motivational tool for increasing awareness of sedentary behavior and are useful for measuring and tracking activity at home or any location [33]. One of the benefits of WATs is that they have the ability to measure a variety of activity-related outcomes, including steps, distance, heart rate, active minutes, calories, and sleep, with high validity and reliability [34,35]. A large systematic review found that using WATs significantly increased the daily step count (*P*<.001), MVPA (*P*<.001), and energy expenditure (*P*=.03) in adult populations [36]. Owing to the rapid advances and relatively low cost of WATs, a growing amount of research has successfully incorporated WATs into interventions to increase PA, reduce obesity, and manage chronic conditions such as breast cancer [22,37]. Results from a qualitative study of breast cancer survivors found that survivors reported acceptance of using WATs, confidence, and comfort in using them, and that using WATs increased their motivation for PA [38]. WATs may also be helpful for promoting PA among cancer patients who are still receiving primary therapy for the disease [39,40]. In addition, WATs have been shown to increase self-awareness of PA and reinforce progress toward meeting PA goals [41]. WATs also show promise as a tool to reduce disparities among patients with cancer and cancer survivors by overcoming barriers such as access to health care providers and health monitoring [42]. WATs are cost-effective, can be widely distributed, have the potential to minimize user burden, and provide immediate feedback in an enjoyable experience for users [43].

Overall, WATs may overcome some limitations of traditional in-person programs for PA and weight management for cancer survivors, such as overcoming travel barriers, decreasing user burden, and addressing time or schedule constraints [30,44,45].

To aid in interpreting the underlying behavior regulations associated with motivation, we examined exercise motivation through the lens of self-determination theory (SDT) [46]. SDT distinguishes between two sources of motivation that regulate a person’s behavior: intrinsic (internal) and extrinsic (external). Intrinsic motivation is defined as engaging in an activity or behavior because of the inherent satisfaction a person gets. An intrinsically motivated person experiences enjoyment, accomplishment, and excitement when engaging in the behavior or action. Extrinsic motivation refers to engaging in a behavior to obtain an outcome outside of what is inherently achieved through doing a behavior. This can include social rewards, such as praise, disapproval avoidance, or monetary incentives.

Furthermore, SDT distinguishes between different types of extrinsic motivation by their style of regulation on behavior. For example, controlled regulation is the least autonomous form of extrinsic motivation. In this regulation style, behavior is primarily driven by externally administered rewards and punishments. Individuals operating from this type of motivation typically experience externally regulated behavior as controlling or alienating, leading to an externally perceived locus of causality or control [47]. In another regulation type, introjected regulation, people will perform actions to avoid feeling guilty or anxious or to satisfy their ego or pride. Although this style is still strongly externally controlled, introjection represents a type of regulation that is also contingent on ego and self-esteem. Although this regulation style is internal to the person, introjected behaviors are not experienced as fully self-determined and still operate from an external locus of control [47]. SDT conceptualizes these motivations as a constant continuum moving between amotivation, or having no motivation, to fully self-determined motivation [46,48]. SDT postulates that meeting goals and changing behavior are more likely to occur if motivation is self-determined or autonomous [24]. Previous studies have demonstrated the efficacy of adapting and mapping SDT concepts to exercise motivations in understanding health behavior [49], particularly mapping guilt onto introjected regulation [48,50,51].

There is still a lot of uncertainty around understanding what motivates cancer survivors to engage in PA. One of the challenges to PA engagement among survivors is that they tend to have lower exercise motivation after diagnosis and treatment [24]. However, some studies have examined exercise motivation among cancer survivors, specifically through the framework of SDT. One study found that breast cancer survivors who meet PA recommendations have higher scores of intrinsic motivation and autonomous regulation, similar to exercise enjoyment as a motivation in this study, than those who did not reach PA guidelines [52]. Other research also indicates that intrinsic motivation is significantly associated with greater long-term exercise adherence [48].

### Objectives

Cancer survivors who meet PA recommendations experience better health outcomes. With the growing availability and implementation of WATs, it may be easier to track PA, but it is still unknown what motivates cancer survivors to wear these devices. Therefore, the purpose of this study is to investigate the relationship among motivations for exercise (internal guilt, pressure from others, physical appearance, and exercise enjoyment), reported previous use of WATs to track health goals, and meeting the recommended amount of PA (≥150 minutes of MVPA per week) among a cohort of cancer survivors.

## Methods

### Data Source

First administered in 2002-2003 by the National Cancer Institute, the Health Information National Trends Survey (HINTS) is a biennial, cross-sectional survey of a nationally representative sample of noninstitutionalized American adults aged 18 years and older that is used to assess the context in which people access and use health information. There are 13 iterations of HINTS, and this study uses the 13th iteration released in January 2020, HINTS 5 Cycle 3, which represents data collected from January to April 2019. Each HINTS iteration has been approved through an expedited review by the Westat Institutional Review Board and deemed exempt by the US National Institutes of Health Office of Human Subjects Research Protections. A total of 5438 people participated in HINTS 5 Cycle 3. In this cycle, the overall response rate was 30.3%. For descriptive analysis, sample weighting was used to provide nationally representative US estimates. The HINTS survey uses weights that are designed to provide population level estimations utilizing a modified Horvitz-Thompson estimator and Jackknife replication method [53].

### Participants

In this study, all cancer survivors who completed a survey for cycle 3 in 2019 with complete information on demographic variables, WAT use, exercise motivation, and minutes of MVPA per week were included (N=608).

### Measures

#### Demographics

Demographic variables included participants’ age (years), BMI, gender (male or female), marital status (married or divorced), household income range, education (less than high school, high school graduate, some college education, college graduate, or more), health insurance status (yes or no), English-speaking proficiency (very well or not very well), self-rated health (excellent, very good, good, fair, or poor), ability to take care of one’s health (completely confident, very confident, somewhat confident, a little confident, or not confident at all), rural or urban designation, cancer type (breast, cervical, prostate, colorectal, skin, other, or more than one type), and time since cancer diagnosis (in years). Race or ethnicity was examined using a dichotomized variable representing survivors from a White racial or ethnic background and those from a non-White racial or ethnic background, including Hispanics, Asians, and African Americans. BMI was used to classify participants as obese (≥30), overweight (29.9-26), or normal weight or underweight (<26).

#### Use of WATs

Participants’ responses to the question, “In the past 12 months, have you used an electronic wearable device to monitor or track your health or activity? For example, a Fitbit, AppleWatch or Garmin Vivofit...” were used to characterize the distribution of subjects who used WATs (yes or no).

#### Exercise Motivation

To assess motivation, we used participants’ responses to questions that asked “Why the participant starts or continues exercise regularly” with separate questions asking if the reason was “pressure from others (external pressure), concern over the way you look (physical appearance), feeling guilty when you stop exercising (internal guilt), or getting enjoyment from exercise (exercise enjoyment).” Answer choices included “A lot,” “Some,” “A little,” or “Not at all.” For regression modeling, we dichotomized the response variable into *not at all* versus *any*.

#### Physical Activity

To investigate the association between WAT use and PA, we created a binary outcome variable derived from a composite of combining responses to questions which asks, “In a typical week, how many days do you do any physical activity or exercise of at least moderate intensity, such as brisk walking, bicycling at a regular pace, and swimming at a regular pace (do not include weightlifting)?” with option choices from *1 day per week* to *7 days per week,* and another question, which asks, “On the days you do physical activity for exercise of at least moderate intensity, how long do you typically do these activities?” and allowed participants to answer with any positive number up to 3 digits in length. To develop the outcome variable, the number of days per week reported was multiplied by the number of minutes to obtain the average time per week of MVPA. We then created a binary variable with either *yes* or *no* options based on whether the participant met recommended weekly minutes of MVPA (yes ≥150 or no <150).

### Statistical Analyses

Before the analysis, data were screened for normality, outliers, and patterns of missing data. Missing data were screened and tested in Statistical Access Software (SAS) version 9.4 using PROC MI to examine the distribution of missing values. No distinct patterns of missing data were found; therefore, the data were approached as missing at random. As no patterns in missing data were found, participants who completed the survey for cycle 3 in 2019 with complete information on demographics, exercise motivations, WAT use, and minutes of moderate-to-vigorous PA (MVPA) per week were included in the study (N=608). Descriptive data for continuous variables were reported as weighted means and SDs, and categorical variables were reported as weighted frequencies and percentages.

To assess the relationship between exercise motivation variables and WAT use, multivariable logistic regression models were used. In addition, we examined the interaction between individual exercise motivations and race or ethnicity to explore differences in motivations by race or ethnicity. A separate multivariable logistic model was used to assess the relationship between WAT use and meeting the recommended amount of PA. A cutoff of *P*<.05 was used to determine statistical significance for all analyses.

A cluster analysis was conducted to generate motivational profiles based on responses to exercise motivation questions using the PROC LCA procedure in SAS 9.4. In PROC LCA, parameters are estimated using an expectation-maximization algorithm to obtain the maximum likelihood. In addition, this procedure incorporates the Newton-Raphson method for the estimation of regression coefficients. The convergence index used in this procedure is the maximum absolute deviation (MAD). The estimation procedure continues to iterate until either a specified criterion value of MAD (the convergence criterion) is met or the maximum number of iterations is reached. Finally, LCA parameter estimates and standard errors are found by inverting the Hessian matrix to obtain the log likelihood [54]. Using this method, we tested the best-fit model as either a 2-, 3-, 4-, or 5-cluster solution. These options were then assessed further using goodness-of-fit statistics, Akaike information criterion, Bayesian information criterion, G-squared, entropy, and interpretability. Once profiles were formed, differences in WAT use were assessed using logistic modeling and chi-square tests. SAS version 9.4 was used for all data modeling and analyses carried out in this study.

## Results

### Demographic Characteristics of the Cohort

Multimedia Appendix 1 describes the cancer cohort. The mean age of the cohort was 66.9 years (SD 12.1), and the mean BMI was 28.3 (SD 6.1). The majority of cancer survivors were non-Hispanic White (473/608, 78.7%), female (322/608, 54.9%), married (328/608, 62.9%), and spoke English very well (546/608, 89.8%). The most frequently reported cancer was skin cancer (154/608, 27.8%), followed by more than one type of cancer (110/608, 18.1%) and breast cancer (79/608, 12.4%), which are among the most prevalent types of cancer in the general population [55]. A large proportion of the cohort completed some college or more (489/608, 71.5%) and frequently reported being in good (228/608, 38.3%) or very good health (194/608, 29.4%) and being very confident that they could take care of their health (279/608, 43.3%). In addition, the cohort overwhelmingly reported having health insurance (596/608, 96.8%). Regarding PA, the majority of this cancer cohort did not meet the recommended amount of PA (396/608, 67.9%) and most only reported between 0 and 74 minutes of MVPA per week (282/608, 49.9%). One-fifth of cancer survivors reported using a WAT device in the past month (119/608, 20.9%). The complete breakdown of exercise motivations reported by WAT users and non-WAT users in provided in Table 1.

**Table 1:** Exercise motivations (wearable activity tracker users vs nonwearable activity tracker users; N=608).

Characteristic and category			WAT^a^ users (n=119), n (%)					Non-WAT users (n=489), n (%)		
			Participants		Participants (weighted)		Participants			Participants (weighted)
**Internal guilt**										
	No	17 (9.6)		330,710 (9.6)		198 (42.9)			5,572,690 (42.9)	
	Yes	102 (90.4)		3,106,554 (90.4)		291 (57.1)			7,422,694 (57.1)	
**Exercise enjoyment**										
	No	20 (12.5)		428,160 (12.5)		123 (23.7)			3,086,204 (23.7)	
	Yes	99 (87.5)		3,009,105 (87.5)		366 (76.3)			9,909,181 (76.3)	
**Physical appearance**										
	No	12 (6.3)		215,926 (6.3)		110 (19.3)			2,503,455 (19.3)	
	Yes	107 (93.7)		3,221,338 (93.7)		379 (80.7)			10,491,930 (80.7)	
**Pressure from others**										
	No	77 (63.6)		2,184,454 (63.6)		323 (67.6)			8,784,210 (67.6)	
	Yes	42 (36.4)		1,252,810 (36.4)		166 (32.4)			4,211,175 (32.4)	

^a^WAT: wearable activity tracker.

### Exercise Motivation and WAT Use—Regression Modeling

When exercise motivations were assessed independently, adjusting for all covariates in a multivariate logistic regression model, cancer survivors who did not report internal guilt as a motivation for exercise were 73% less likely to use WATs (odds ratio [OR] 0.27, 95% CI 0.14-0.54; *P*<.001). This model was adjusted by participant’s age, BMI, time since cancer diagnosis, gender, marital status, household income range, level of educational attainment, race or ethnicity, self-rated health, self-efficacy for health, region, urban or rural status, health insurance status, English-speaking ability, and type of cancer diagnosis. In addition, several demographic variables were found to be significantly associated with WAT use in this model. An increase in age was associated with a decreased likelihood of using WATs (OR 0.95, 95% CI 0.93-0.97; *P*<.001). In addition, survivors with higher income (US $75,000-$199,000 vs US $0-$34,000; OR 2.84, 95% CI 1.22-6.59; *P*=.02) and those with better health (fair or poor vs excellent; OR 0.2, 95% CI 0.07-0.61; *P*=.004) were more likely to use WATs. The time since cancer diagnosis was included as a control variable in this model and was found to be not statistically significantly associated with WAT use (*P*=.93). Finally, when testing for interactions between individual exercise motivations and race or ethnicity, we found no significant interactions. The results are presented in Table 2.

**Table 2:** Results from multivariable regression modeling of exercise motivations and previous wearable activity tracker use (N=608).

Variable^a^	Odds ratio (95% CI)	*P* value
Pressure from others^b^	1.17 (0.70-1.97)	.54
Physical appearance^c^	0.67 (0.30-1.53)	.35
Internal guilt^b^	0.27 (0.14-0.54)	<.001
Exercise enjoyment^c^	0.82 (0.40-1.60)	.55
Age	0.95 (0.93-0.97)	<.001
Income^d^	2.84 (1.22-6.49)	.02
Self-rated health^e^	0.20 (0.07-0.61)	.004

^a^Adjusted for age, BMI, time since cancer diagnosis, gender, marital status, household income range, level of educational attainment, race or ethnicity, self-rated health, self-efficacy for health, region, urban or rural status, health insurance status, English-speaking ability, and type of cancer diagnosis.

^b^None versus any motivated.

^c^Any versus not motivated.

^d^US $75,000-$199,000 versus US $0-$34,000.

^e^Fair or poor versus excellent.

### Exercise Motivation and WAT Use—Cluster Analysis

**Figure 1:**
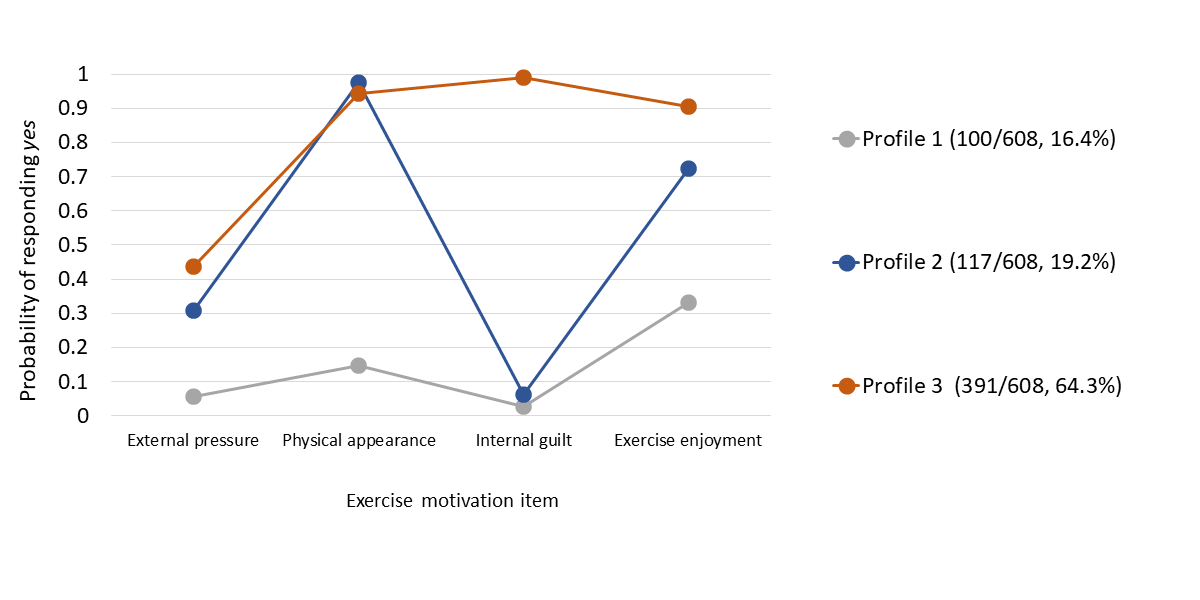
Latent class analysis of motivation profiles (N=608), adjusting for age.

Figure 1 displays the 3 motivational profiles that emerged from the cluster analysis. The profiles differed significantly across motivation and class membership.

Profile 1 (100/608, 16.4%) is characterized by cancer survivors who did not report being influenced to exercise by any of these motivations (internal guilt, pressure from others, physical appearance, and exercise enjoyment).

Profile 2 (117/608, 19.2%) profile is characterized by cancer survivors who reported exercising because of exercise enjoyment (intrinsic motivation with autonomous regulation) and physical appearance (extrinsic motivation with introjected regulation).

Profile 3 (394/608, 64.4%) is characterized by cancer survivors who reported being motivated by exercise enjoyment (intrinsic with autonomous regulation) and strongly by both physical appearance and internal guilt (extrinsic motivation with introjected regulation).

WAT users had an 86% probability of membership in profile 3 (gamma=0.86; SE 0.04; *P*<.001) versus profile 1, whereas non-WAT users only had a 58% (gamma=0.58; SE 0.04; *P*<.001) chance of being in this profile. When assessed in a logistic regression model, profile 3 was also the only cluster that was significantly associated with WAT use (OR 4.5, 95% CI 2.1-9.7; *P*<.001) after adjusting for participants’ age, BMI, time since cancer diagnosis, gender, marital status, household income range, level of educational attainment, race or ethnicity, self-rated health, self-efficacy for health, region, urban or rural status, health insurance status, English-speaking ability, and type of cancer diagnosis.

### Association Between WAT Use and PA

Cancer survivors who used WATs were 1.6 times more likely to meet PA recommendations than those who did not use WATs (OR 1.65, 95% CI 1.03-2.65; *P*=.04). In addition, in this model, we found that survivors who had lower BMI (OR 0.92, 95% CI 0.89-0.96; *P*<.001), had higher household income (US $200,000+ vs US $0-$35,000; OR 2.62, 95% CI 1.11-6.19; *P*=.03), and were in better health (fair or poor vs excellent; OR 0.18, 95% CI 0.07-0.44; *P*<.001) were more likely to meet weekly PA recommendations. The results can be found in Table 3.

**Table 3:** Association between wearable activity tracker use and meeting the recommended amount of physical activity (N=608).

Variable^a^	Odds ratio (95% CI)	*P* value
Previous wearable activity tracker use^b^	1.65 (1.03-2.65)	.04
BMI	0.92 (0.89-0.96)	<.001
Household income^c^	2.62 (1.11-6.19)	.03
Self-rated health^d^	0.18 (0.07-0.44)	<.001

^a^Adjusted for age, BMI, time since cancer diagnosis, gender, marital status, household income range, level of educational attainment, race or ethnicity, self-rated health, self-efficacy for health, region, urban or rural status, health insurance status, English-speaking ability, and type of cancer diagnosis.

^b^Yes versus no wearable activity tracker use.

^c^US $200,000+ versus US $0-$35,000.

^d^Fair or poor versus excellent.

## Discussion

### Principal Findings

One of our primary objectives was to examine the associations of internal guilt, exercise enjoyment, pressure from others, and physical appearance as motivations for exercise with reporting having used WATs to track a health goal among a cohort of cancer survivors. The second objective was to examine clusters of exercise motivations associated with reporting previous WAT use. When exercise motivations were assessed independently, only internal guilt was significantly associated with WAT use among this cohort of cancer survivors. However, in the cluster analysis, 3 distinct motivational profiles emerged with distinctly different class memberships. WAT users were significantly more likely to be in profile 3, a group characterized by being motivated by internal guilt, physical appearance, and exercise enjoyment (autonomous with high introjected regulation). The cluster analysis provided a unique examination on not only how a single exercise motivation is associated with reporting WAT use but also how a combination of motives can be identified.

In both analyses, external guilt as a motivation for exercise emerged as being significantly associated with reporting previous WAT use. There is concern that guilt as a motivation can be harmful to healthy behavior adherence and that using WATs can cause additional stress or induce negative affect [56]. However, in this study, we observed a significant relationship between health-related internal guilt and reporting using WATs to track a health goal or activity. Health-related guilt in this context is a negative feeling about a person’s own behavioral shortcomings related to health, often through self-blame. For example, a person may feel guilty when they have not exercised, although having been given recommendations from a health provider to do so. This experience typically involves a sense of anxiety or regret [50]. However, the experience of guilt is typically in response to a specific behavior, unlike shame, which is a negative feeling about oneself or global self-blame. Therefore, the experience of guilt is typically less painful than shame [50]. This may explain why previous studies have found an association between guilt and higher levels of MVPA among breast cancer survivors [51].

Understanding exercise motivation through a framework of SDT helps us to identify and differentiate sources of exercise motivation (internal vs external) and allows us to conceptualize different forms of control or behavior regulation within extrinsic motivation (eg, introjected regulation and controlled regulation). In this context, we can think of health-related guilt as an emotion. However, considering the underlying behavior regulation associated with guilt, we apply an SDT framework, specifically mapping guilt onto extrinsic motivation with introjected regulation [48,50,51].

Understanding the type of behavioral regulation linked with guilt can inform the planning and design of technology-based mobile health (mHealth) interventions that focus on addressing the behavioral regulation aspect of health-related guilt while not directly leveraging or increasing the emotional aspect that may negatively impact healthy behavior adherence.

Given that motivation in the context of SDT exists on a continuum, viewing the results of this study through an SDT framework can potentially inform the development of interventions that focus on moving survivors from extrinsically motivated regulations such as introjected regulation (eg, guilt) to more autonomous forms of motivational control (eg, enjoyment). One approach is to design intervention components such as motivational messages that avoid guilt- or shame-inducing language and instead aid the user in becoming more accountable for meeting MVPA recommendations while creating enjoyable experiences. This can potentially be achieved by using mHealth intervention components such as gamification and motivational affordances (eg, leaderboards, badges, and challenges), which help to foster more autonomous forms of regulation and motivation (eg, enjoyment and mastery). Clinicians may also play a role in guiding their patients toward making more positive cognitive appraisals directed at managing feelings of guilt. This process distinguishes between health-related guilt and engaging in self-blaming behavior (eg, failure and shame), which has been found to be associated with negative health consequences and decreased PA motivation [51].

On the basis of these findings, motivational regulation is likely to be an important factor linking body-related emotions and MVPA. WAT interventions typically contain behavior change techniques that include monitoring and tracking but rarely address extrinsic motivation with introjected regulation (eg, guilt). There is a need to recognize that health- and body-related guilt exists among cancer survivors and consider the implications of the relationship between guilt and health behaviors among this population.

Another objective of this study is to examine the association between WAT use and meeting the recommended amount of weekly MVPA among this cohort of cancer survivors. Reporting previous WAT use for tracking health goals was statistically significantly associated with meeting MVPA recommendations. Given the health benefits of PA for cancer survivors and the potential barriers to in-person PA programs, interventions aimed at aiding cancer survivors in meeting MVPA recommendations could leverage WATs to help survivors reach these goals.

### Comparison With Previous Work

Similar to previous findings, we found that enjoyment (intrinsic motivation), a more autonomous form of behavioral regulation, was found to play a role in reporting WAT use when looking at clusters of motivation [57]. However, contrary to previous work, we did not find that external pressure from others to exercise was associated with WAT use [58].

Although previous studies have investigated the relationships among demographic, health, and lifestyle variables associated with meeting PA guidelines in cancer survivors, few have investigated the role of reporting previous WAT use in meeting PA guidelines among cancer survivors [49]. A large systematic review found that cancer survivors showed an increase in PA when using WATs and that increased PA played an important role in alleviating the adverse health effects of breast cancer therapy [22]. Another study found that WATs motivated breast cancer survivors to be physically active and created more awareness of their sedentary lifestyle [37]. Results from a qualitative study found similar findings in that WATs increased self-awareness and motivation among breast cancer survivors [38].

### Future Considerations

Findings from this study can provide insights into the relationship between reporting internal guilt as an exercise motivation and reporting meeting MVPA recommendations for cancer survivors. The results can also provide some insights into possible ways to interpret guilt as an exercise motivation and potentially understand the underlying behavior regulation of this emotion through a framework of SDT. There remains an opportunity for future researchers to address questions regarding the intensity of WAT use among cancer survivors and the amount of PA. There also remains uncertainty as to whether WATs act as a facilitator of PA or a primary driver of health behavior [59]. In addition, there are technological difficulties to consider (initial setup, troubleshooting, etc) that can create barriers to PA adherence in home-based PA interventions among cancer survivors [59]. In addition, there is concern that WATs can cause stress or induce negative effects on healthy behavior, which can also be problematic [56]. However, studies have shown successful integration of WATs into interventions with no reported increase in negative affect or causing unwanted stress [60]. This study will also serve to inform a follow-up paper focused on the intensity of WAT use, exercise motivation, and PA.

### Limitations

Although HINTS is designed to be nationally representative, the data were collected through a self-report, cross-sectional survey. Thus, we are unable to analyze trends in WAT use, motivations, and PA over time and must rely on a person’s recollection of events and behaviors. In addition, because this is a cross-sectional survey, we were limited to the questions and variables that were included in the survey, such as being limited to examining only the range of the exercise motivations included in the survey and being unable to know what specific health measures or activities the participants were tracking on their wearable devices. There is also the possibility of unmeasured confounding, which might be associated with mHealth engagement that would influence the interpretation of these results. Although our analyses showed a statistically significant association, it does not indicate a causal relationship, and we cannot address the issue of temporality, given the cross-sectional nature of the study. For example, we cannot determine whether a motivation leads to WAT use or if WAT use leads to motivation. Our goal was to determine associations among motivations for exercise, WAT use, and meeting PA recommendations among this cohort of cancer survivors; thus, our results should not be generalized to populations outside of survivors. Finally, because of smaller data cell counts, we had to examine interactions for race using a dichotomized variable derived from cancer survivors reporting if they were from a White racial or ethnic background or if they were from a non-White racial or ethnic background. Due to this dichotomization, we may have been unable to detect more subtle but significant differences in motivations by race. Finally, we need to consider that those who used WATs had more access to devices based on higher socioeconomic status (SES) and must consider the implications for cancer survivors with lower SES. Although this study was a secondary analysis of cross-sectional data, the results add to the literature supporting the notion that previous WAT use among cancer survivors is associated with reported meeting MVPA guidelines.

### Conclusions

When assessed individually, internal guilt as an exercise motivation (extrinsic motivation with introjected regulation) was found to be significantly associated with reporting previous WAT use among a cohort of cancer survivors. In a cluster analysis, WAT users were more likely to be in a profile that reported being motivated to exercise by internal guilt, exercise enjoyment, and physical appearance, demonstrating a combination of intrinsic and extrinsic motivations (autonomous with high introjected regulation). This provides us with insights on not only how one motivation but how a confluence of motivations was found to be associated with reporting previous WAT use for tracking health goals among a cohort of cancer survivors. However, in both analyses, we found that internal guilt was consistently reported as an exercise motivation associated with reported WAT use. We can also apply an SDT framework to better understand the underlying behavioral regulation that underlies health-related guilt. In addition, among this cohort of cancer survivors, WAT use was significantly associated with meeting the PA recommendation guidelines. The results of this study can aid in identifying which cancer survivors are more or less likely to use WATs and the potential underlying motivations and behavior regulations that are associated with their use. Given the health benefits of PA for cancer survivors, technology-focused interventions targeting exercise motivation may aid cancer survivors in meeting MVPA recommendation guidelines.

## Appendix

Multimedia Appendix 1 Descriptive characteristics of the cancer cohort (wearable activity tracker users vs nonwearable activity tracker users; N=608).

## Abbreviations

HINTS: Health Information National Trends Survey
MAD: maximum absolute deviation
mHealth: mobile health
MVPA: moderate-to-vigorous physical activity
OR: odds ratio
PA: physical activity
SDT: self-determination theory
SES: socioeconomic status
WAT: wearable activity tracker
