# Supplementary material for: Associations Among Wearable Activity Tracker Use, Exercise Motivation, and Physical Activity in a Cohort of Cancer Survivors: Secondary Data Analysis of the Health Information National Trends Survey
Source: JMIR Cancer. 2021 Apr 12;7(2):e24828. doi: 10.2196/24828 (PMC8076994; doi:10.2196/24828)
Supplement: Multimedia Appendix 1 [file cancer_v7i2e24828_app1.pdf]

Characteristic and category		WAT[a] users (n=119)			Non-WAT users (n=489)		
		Participants, n (%)	Participants (weighted), n (%)	Value, mean (SD)	Participants, n (%)	Participants (weighted), n (%)	Value, mean (SD)
**Age (years)**		—[b]	—	62 (12)	—	—	68 (12)
	18-34	3 (7.8)	267,423 (7.8)	—	5 (1.9)	248,523 (1.9)	—
	35-49	16 (18.1)	620,483 (18.1)	—	28 (10.2)	1,328,865 (10.2)	—
	50-64	49 (41.3)	1,418,560 (41.3)	—	131 (33.7)	4,373,718 (33.7)	—
	65-74	38 (25.3)	871,232 (25.3)	—	174 (26.8)	3,485,711 (26.8)	—
	75+	13 (7.6)	259,566 (7.6)	—	151 (27.4)	3,558,568 (27.4)	—
**BMI**		—	—	28 (6)	—	—	28 (6)
	Underweight (>18.5)	0	0	—	8 (1.6)	202,794 (1.6)	—
	Normal (18.5-24.9)	37 (31.1)	1,068,462 (31.1)	—	142 (26.5)	3,441,782 (26.5)	—
	Overweight (25-29.0)	47 (47.0)	1,614,218 (47.0)	—	177 (37.5)	4,873,882 (37.5)	—
	Obese (30.0 +)	35 (22.0)	754,585 (22.0)	—	162 (34.5)	4,476,927 (34.5)	—
**Gender**							
	Male	48 (44.7)	1,536,168 (44.7)	—	238 (45.2)	5,873,844 (45.2)	—
	Female	71 (55.3)	1,901,097 (55.3)	—	251 (54.8)	7,121,541 (54.8)	—
**Education**							
	High school or less	9 (15.2)	523,682 (15.2)	—	110 (32.0)	4,161,330 (32.0)	—
	Some college	33 (39.8)	1,368,747 (39.8)	—	154 (39.8)	5,172,365 (39.8)	—

	College graduate or higher	77 (44.9)	1,544,836 (44.9)	—	225 (28.2)	3,661,690 (28.2)	—
**Income ranges (household)**							
	US $0-$34,999	11 (10.9)	374,294 (10.9)	—	166 (35.2)	4,577,416 (35.2)	—
	US $35,000-$74,999	46 (42.5)	1,462,366 (42.5)	—	169 (31.8)	4,133,740 (31.8)	—
	US $75,000-$199,000	47 (33.8)	1,163,258 (33.8)	—	125 (26.7)	3,466,481 (26.7)	—
	US $200,000 +	15 (12.7)	437,347 (12.7)	—	29 (6.3)	817,748 (6.3)	—
**Self-rated health**							
	Fair or poor	9 (13.6)	467,740 (13.6)	—	125 (27.1)	3,515,438 (27.1)	—
	Good	42 (30.3)	1,042,569 (30.3)	—	186 (40.4)	5,245,298 (40.4)	—
	Very good	46 (37.7)	1,294,532 (37.7)	—	148 (27.2)	3,534,574 (27.2)	—
	Excellent	22 (18.4)	632,423 (18.4)	—	30 (5.4)	1,249,725 (5.4)	—
**Self-efficacy for health**							
	Completely confident	47 (35.8)	1,231,582 (35.8)	—	102 (22.0)	3,433,266 (22.0)	—
	Very confident	51 (42.7)	1,467,989 (42.7)	—	228 (43.5)	5,658,152 (43.5)	—
	Not very confident	21 (21.5)	737,694 (21.5)	—	159 (34.5)	3,853,136 (34.5)	—
**English-speaking proficiency**							
	Not very well	10 (9.8)	335,638 (9.8)	—	52 (10.3)	1,336,090 (10.3)	—
	Very well	109 (90.2)	3,101,626 (90.2)	—	437 (89.7)	11,659,294 (89.7)	—
**Health insurance status**							
	Yes	117 (99.5)	3,421,303 (99.5)	—	479 (96.1)	12,491,744 (96.1)	—
	No	2 (0.5)	15,962 (0.5)	—	10 (3.9)	503,641 (3.9)	—
**Marital status**							
	Unmarried	43 (30.9)	1,061,722 (30.9)	—	237 (38.8)	5,035,783 (38.8)	—

	Married	76 (69.1)	2,375,542 (69.1)	—	252 (61.2)	7,959,601 (61.2)	—
**Rural urban designation**							
	Metro	109 (88.2)	3,031,954 (88.2)	—	440 (86.9)	11,298,942 (86.9)	—
	Urban	8 (7.6)	260,067 (7.6)	—	40 (9.0)	1,164,385 (9.0)	—
	Rural	2 (4.2)	145,243 (4.2)	—	9 (4.1)	532,057 (4.1)	—
**Region**							
	Northeast	14 (11.1)	380,958 (11.1)	—	56 (19.4)	2,520,639 (19.4)	—
	Midwest	22 (20.9)	717,840 (20.9)	—	96 (20.1)	2,607,081 (20.1)	—
	South	54 (44.3)	1,522,627 (44.3)	—	233 (42.1)	5,471,447 (42.1)	—
	West	29 (23.7)	815,840 (23.7)	—	104 (18.4)	2,396,218 (18.4)	—
**Cancer type**							
	More than one type	20 (21.0)	722,155 (21.0)	—	90 (17.4)	2,259,751 (17.4)	—
	Other	27 (23.4)	804,213 (23.4)	—	118 (25.2)	3,274,579 (25.2)	—
	Skin	28 (22.5)	771,734 (22.5)	—	126 (29.2)	3,794,037 (29.2)	—
	Colorectal	7 (6.3)	216,424 (6.3)	—	29 (5.6)	727,184 (5.6)	—
	Prostate	9 (6.9)	237,907 (6.9)	—	50 (6.9)	900,086 (6.9)	—
	Cervical	9 (5.2)	177,916 (5.2)	—	16 (4.0)	513,807 (4.0)	—
	Breast	19 (14.7)	506,917 (14.7)	—	60 (11.7)	1,525,941 (11.7)	—
**Time since diagnosis (years)**							
	<1	18 (18.1)	622,968 (18.1)	—	61 (16.5)	2,148,143 (16.5)	—
	2-5	25 (20.8)	715,968 (20.8)	—	105 (23.3)	3,027,287 (23.3)	—
	6-10	24 (11.3)	387,202 (11.3)	—	85 (17.1)	2,220,028 (17.1)	—
	11+	52 (49.8)	1,711,126 (49.8)	—	238 (43.1)	5,599,927 (43.1)	—

Weekly minutes of MVPA[c]		—	—	207 (215)	—	—	151 (298)
**Physical activity category**							
	0-74 min per week	36 (38.7)	1,329,721 (38.7)	—	246 (52.8)	6,861,949 (52.8)	—
	75-149 min per week	22 (18.5)	636,704 (18.5)	—	92 (18.0)	2,343,491 (18.0)	—
	150-300 min per week	35 (18.9)	649,155 (18.9)	—	94 (15.8)	2,054,179 (15.8)	—
	300+ min per week	26 (23.9)	821,684 (23.9)	—	57 (13.4)	1,735,766 (13.4)	—
**Meeting recommended amount of physical activity**							
	No	58 (57.2)	1,966,425 (57.2)	—	338 (70.8)	9,205,440 (70.8)	—
	Yes	61 (42.8)	1,470,839 (42.8)	—	151 (29.2)	3,789,945 (29.2)	—

[a]WAT: wearable activity tracker.

[b]Not applicable.

[c]MVPA: moderate-to-vigorous physical activity.

This is a Multimedia Appendix to a full manuscript published in the J Med Internet Res. For full copyright and citation information see http://dx.doi.org/10.2196/jmir.24828
